# Distributed, high-resolution modelling of critical source areas for erosion and phosphorus losses

**DOI:** 10.1007/s13280-014-0618-4

**Published:** 2015-02-15

**Authors:** Faruk Djodjic, Ana Villa

**Affiliations:** Department of Aquatic Sciences and Assessment, Swedish University of Agricultural Sciences, Lennart Hjälmsv. 9, P.O. Box 7050, 75007 Uppsala, Sweden

**Keywords:** Erosion, Modelling, Phosphorus, Eutrophication

## Abstract

Phosphorus losses from arable land need to be reduced to prevent eutrophication of surrounding waters. Owing to the high spatial variability of P losses, cost-effective countermeasures need to target parts of the catchment that are most susceptible to P losses. Field surveys identified critical source areas for overland flow and erosion amounting to only 0.4–2.6 % of total arable land in four different catchments in southern Sweden. Distributed modelling using high-resolution digital elevation data identified 72–96 % of these observed erosion and overland flow features. The modelling results were also successfully used to predict occurrence of overland flow and rill and gully erosion in a catchment in central Sweden. Such exact high-resolution modelling allows for accurate placement of planned countermeasures. However, current legislative and environmental subsidy programmes need to change their approach from income-loss compensation to rewarding high cost effectiveness of implemented countermeasures.

## Introduction

Human activities, including modern agriculture, distort the nitrogen (N) cycle and phosphorus (P) flows and have altered the status of lake and marine ecosystems (Rockström et al. 2009). According to Carstensen et al. (2014), there has been a 10-fold increase in hypoxia in the Baltic Sea, and this is primarily linked to increased inputs of nutrients from land. Thus, stringent nutrient reductions will be necessary to reduce the impacts of deoxygenation on ecosystems. Achieving good ecological status for inland waters according to the EU Water Framework Directive and the ambitious Country Allocated Reduction Targets for the Baltic Sea agreed at the HELCOM Copenhagen Ministerial Meeting (HELCOM 2013) will demand further reductions in P transfer from terrestrial systems in general and from agriculture in particular.

While losses of N are generally less scale dependent and more management related, the majority (~80 %) of P losses originate from a small proportion of catchment area (~20 %), a situation known as the 80:20 rule (Sharpley et al. 2009). These critical source areas (CSAs) coincide with hydrologically active, interconnected areas where overland and/or shallow subsurface flow mobilize and transfer P from terrestrial to aquatic ecosystems (Pionke et al. 2000). These CSAs are spatially variable over the watershed and even within individual fields, so differing management levels are appropriate for different areas of the watershed (Gburek et al. 2000).

Precise identification of CSAs is therefore a precondition for cost-effective abatement strategies. Targeting CSAs with suitable Best Management Practices (BMP) can lead to both increased removal efficiency and reduced implementation costs. An example of this is grass buffer strips, which are a widely adopted countermeasure in Europe and the USA (Dorioz et al. 2006). Grass buffer strips are inserted between agricultural fields and surface water bodies with the main aim of limiting the delivery of suspended solids (SS) and P from source (field) to recipient (water course or lake). In Sweden alone, there were 11 520 ha of such buffer strips in 2012 (The Environmental Objectives Portal 2014). Based on a review of 11 field studies, Dorioz et al. (2006) concluded that grass buffer strips are able to limit significantly (>50 % retention) the transfer to surface water of sediment and total-P. Considering such high retention, the cost effectiveness of buffer strips is mainly determined by the amounts and concentrations of incoming pollutants. In other words, appropriately placed buffer strips that intercept overland flow should be a cost-effective countermeasure, whereas buffer strips located in areas with no or limited overland flow will be highly ineffective, and therefore very expensive.

As an example, under Swedish Board of Agriculture regulations, farmers are entitled to subsidies for buffer strips in certain areas of the country, mainly in southern Sweden. The buffer strips must be at least 6 m wide, and the parcels length along water body must be at least 20 m, but there are no requirements regarding their placement in the landscape. In addition, the subsidy is fixed, based on buffer strip area and intended to compensate farmers for loss of income rather than to reward higher nutrient retention. Considering the spatial variability in overland flow, this means that large parts of buffer strips never receive any overland flow and therefore can never fulfil their main purposes of retention of sediment and P.

Based on this, identification of hydrologically active areas with obvious traces of overland flow and erosion is the first step in appropriate placement of a certain countermeasure in order to maximize its cost effectiveness.

Topography exerts first-order control on spatial variations in hydrological conditions (Sørensen et al. 2006). Digital terrain analysis is a geographic information system tool that allows users to describe landscapes geospatially in a hydrological, biological or geomorphological context (Galzki et al. 2011). Increasing availability of high-resolution, highly accurate digital elevation models (DEMs) due to advances in light detection and ranging technologies allow for accurate representation of landscape topography and hydrology, with the increasing potential to accurately identify the spatial distribution of processes such as overland runoff and erosion. The Universal Soil Loss Equation (Wischmeier and Smith 1978) and the Revised Universal Soil Loss Equation (Renard et al. 1991) are empirical equations for the computation of soil losses in agricultural fields. By considering the influence of flow convergence or divergence (Mitasova et al. 1996) on erosion/deposition processes and replacing slope length (L) and steepness (S) factors with upslope contributing area (Moore and Burch 1986), the modified Unit Stream Power Erosion Deposition (USPED) model utilizes the accuracy of high-resolution DEM to predict the spatial distribution of erosion processes across the watershed.

The main objectives of the present study were to (1) evaluate the potential of the modified USPED model in combination with high-resolution DEM to identify erosion and overland flow prone areas by comparison with independent field surveys, and (2) evaluate possibilities to use such modelling results to design monitoring and abatement strategies.

## Materials and methods

### Study areas

**Fig. 1:**
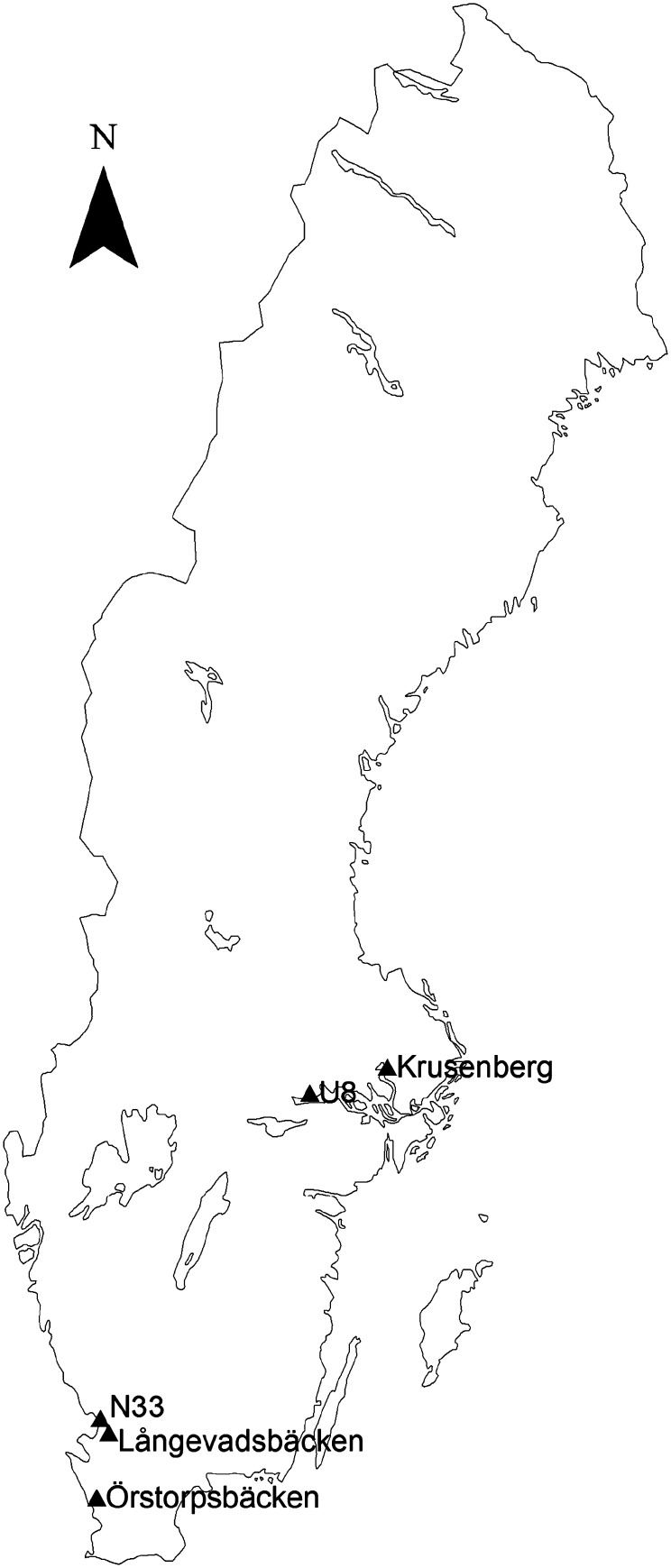
Location of the catchments in Sweden

In total, five catchments were used as study areas (Fig. 1). They are all situated in southern Sweden and vary in sizes ranging from 5.7 to 41.2 km^2^. They represent a range of climate, land use and soil-type conditions (Table 1), although agricultural land occupies a large proportion of each catchment (39–89 %).

**Table 1:** Characteristics of the five catchments studied

Catchment	County	Area (km^2^)	Agriculture (%)	Dominant soil texture class	Temp. (°C)	Precipitation (mm)	Water discharge (mm)
Örstorpsbäcken	Skåne	15.7	89	Loam	8.1	767	245
Långevadsbäcken	Halland	15.2	74	Silt loam	7.6	849	409
N33	Halland	6.6	86	Loam	7.3	772	288
U8	Västmanland	5.7	53	Clay	6.0	539	250
Krusenberg	Uppsala	41.2	39	Clay	6.2	588	187

Field surveys with identification and mapping of surface runoff and erosion-prone areas were conducted within four catchments. In a recent report (Ekologgruppen i Landskrona AB 2010), traces of overland flow and rill and gully erosion were documented through a combination of field surveys and evaluation of high-resolution aerial photographs of “Örstorpsbäcken” and “Långavedsbäcken”. Kyllmar et al. (2013) reported observations of overland flow, erosion, and ponded water in catchments “N33” and “U8” gathered from various sources, including farmers’ observations and field surveys conducted by local authorities and farm advisory services. All observations were marked on paper maps and later digitalized. The field surveys were carried out during early spring, since most overland flow and erosion in Sweden occurs during this period. Snowmelt in particular is a crucial factor for erosion losses (Brandt 1990).

The fifth catchment, “Krusenberg”, situated in the vicinity of the Uppsala was used as a pilot catchment to study the possibility of using model results as guidance for identification of erosion rills and gullies after high-flow episodes. The modelling of erosion pathways in this catchment was performed in October 2012, and repeated field surveys were carried out in winter (December 2012) and spring (April 2013) to verify the existence of overland flow, erosion rills and gullies. The magnitude of sediment and P losses from the most vulnerable field were estimated by field measurements, analyses of high-resolution (0.25 m) orthophoto images, soil sampling and analyses of soil P content.

### Input data, modelling and evaluation

The base layer for the modelling work was a DEM in raster format. A 2-m grid based on the light detection and ranging data was used, with a density of 0.5–1 point m^−2^ and accuracy which is usually better than 0.1 m (Lantmäteriet 2014). The modified USPED model (Mitasova et al. 2001) was implemented within a frame of PCRaster software for environmental modelling (Schmitz et al. 2009). In short, USPED is a simple model which predicts the spatial distribution of erosion and deposition patterns based on the change in overland flow depth and on the local geometry of terrain, including both profile and tangential curvatures. The slope length factor of the revised universal soil loss equation is replaced with upslope contributing area in the modified model, and the LS factor is calculated according to

1 $$ LS = \left( {\frac{A}{22.13}} \right)^{1.6 } \times\, (\sin b)^{1.3} , $$

where *A* is upslope contributing area, and *b* is the slope angle. Exponent values of 1.6 and 1.3 were used here, as recommended by Mitasova et al. (2001). The catchment-specific mean annual runoff (Table 1) was used as the rainfall erosivity factor (*R*). The values of soil erodibility factor (*K*) were based on the new soil map of Swedish agricultural soils (Swedish Board of Agriculture 2014a), in combination with soil maps from the Geological Survey of Sweden for non-agricultural areas. Each textural soil class was assigned a specific K value according to Stone and Hilborn (2012). Land use map and cover factor (*C*) values from Stone and Hilborn (2012) were combined to spatially distribute effects of vegetation cover. Since the aim of the modelling was to compare and rank relative long-term erosion and overland flow risk, all arable soil was assigned the same cover factor (*C*) representative for cereal crops (*C* = 0.35), without consideration of actual crop distribution.

Slope profile and tangential curvature calculated from DEM were used to account for the effect of slope form on erosion and deposition patterns. Uniform, nose and convex linear slopes yield more sediment than concave linear and head slopes, where sediment is deposited on toe slopes (Rieke-Zapp and Nearing 2005). To account for these patterns, the erosion was calculated as

2 $$ A = R \times {\text{LS}} \times C \times K \times \left( {1 + - 1 \times {\text{PC}}} \right) \times \left( {1 + - 1 \times {\text{TC}}} \right), $$

where PC is profile curvature and TC is tangential curvature. According to Eq. 2, convex parts of the landscape (negative profile curvature) are assigned positive values, indicating net erosion, while concave parts of the landscape (positive profile curvature values) are assigned negative values, indicating net deposition. The same approach applies for the tangential curvature: according to Eq. 2, positive values of tangential curvature (laterally convex, resulting in diversion of flow) are assigned negative values, indicating net deposition, whereas negative values of tangential curvature (laterally concave, resulting in concentration of flow) are assigned positive values, indicating net erosion. Consequently, each grid cell is assigned a positive net erosion value or negative net deposition value. Finally, in the last step, the *accuflux* operation in PCRaster is used to calculate for each cell the accumulated amount of material that flows out of the cell into its neighbouring downstream cell. This accumulated value is the amount of material in the cell itself, plus the amount of material in upstream cells of the cell. The local drain direction network, with flow directions from each cell to its steepest downslope neighbour, based on high-resolution DEM is used to accumulate eroded material along flow paths. In our case, flow accumulation along parts of the landscape with positive net erosion cells resulted in increasing erosion. In contrast, flow accumulation along parts of the landscape with negative net deposition values decreased erosion, due to deposition of material.

As the main objective was to identify erosion- and overland flow-prone areas on arable land, the results obtained in erosion modelling were post-processed to separate and visualize the subareas of agricultural land most prone to overland flow and erosion. Using the “Slice” tool with the “Equal Area” method and 50 output zones within ArcGIS 10.2.1 (©1999–2013 Esri Inc.), 2-m grid cells were reclassified and ranked according to modelled erosion vulnerability. Such approach allows incremental identification of CSAs starting with the top 2 % of total agricultural area with the highest erosion values according to modelling results, and thereafter, if necessary, stepwise 2 %-increase.

Thereafter, these 2 % top-ranked cells were compared with observed areas of overland flow and erosion using the “Selection by location” tool within ArcGIS 10.2.1 (©1999–2013 Esri Inc.), which identified all observed areas that intersected with the modelled areas.

### Soil sampling and analyses

In order to quantify P mobilization from the most vulnerable field in the “Krusenberg” catchment, five soil samples were collected in the immediate vicinity of an obvious gully. Four of the samples were collected from the topsoil in the vicinity of the gully and the fifth from the bottom of the gully. Each sample (10 cm deep) was composed of 15 soil cores collected from an area of 2 m^2^. The soils were air-dried, gradually broken down by hand and sieved before analysis for soil dispersion (<5 mm), content of plant-available P and soil total P (TP). The risk of sediment and P mobilization was estimated with the DESPRAL test, performed as described by Withers et al. (2007). SS, TP and dissolved P (DP) were determined in DESPRAL aliquots in accordance with methods issued by the European Committee for Standardization (ECS 1996). Total phosphorus was analysed as soluble molybdate-reactive P after digestion in an acid persulphate solution, DP was determined on filtered samples using flow injection analysis, SS was determined by filtration through 0.2-µm pore membrane filters as the increase in filter weight, and unreactive P (UP) was calculated as the difference between TP and DP. Turbidity was also measured on post-dispersion aliquots using a Hach 2100AN instrument (Hach Company, CO) and expressed as nephelometric turbidity units.

Plant-available soil P was determined by extraction with ammonium lactate/acetic acid (P-AL) at pH 3.75 (Egnér et al. 1960), which is the standard agronomic soil P test in Sweden. Soil TP was determined by extraction with acid digestion following the Swedish standard method SS 28311 (Swedish Standards Institute 1997).

## Results

**Fig. 2:**
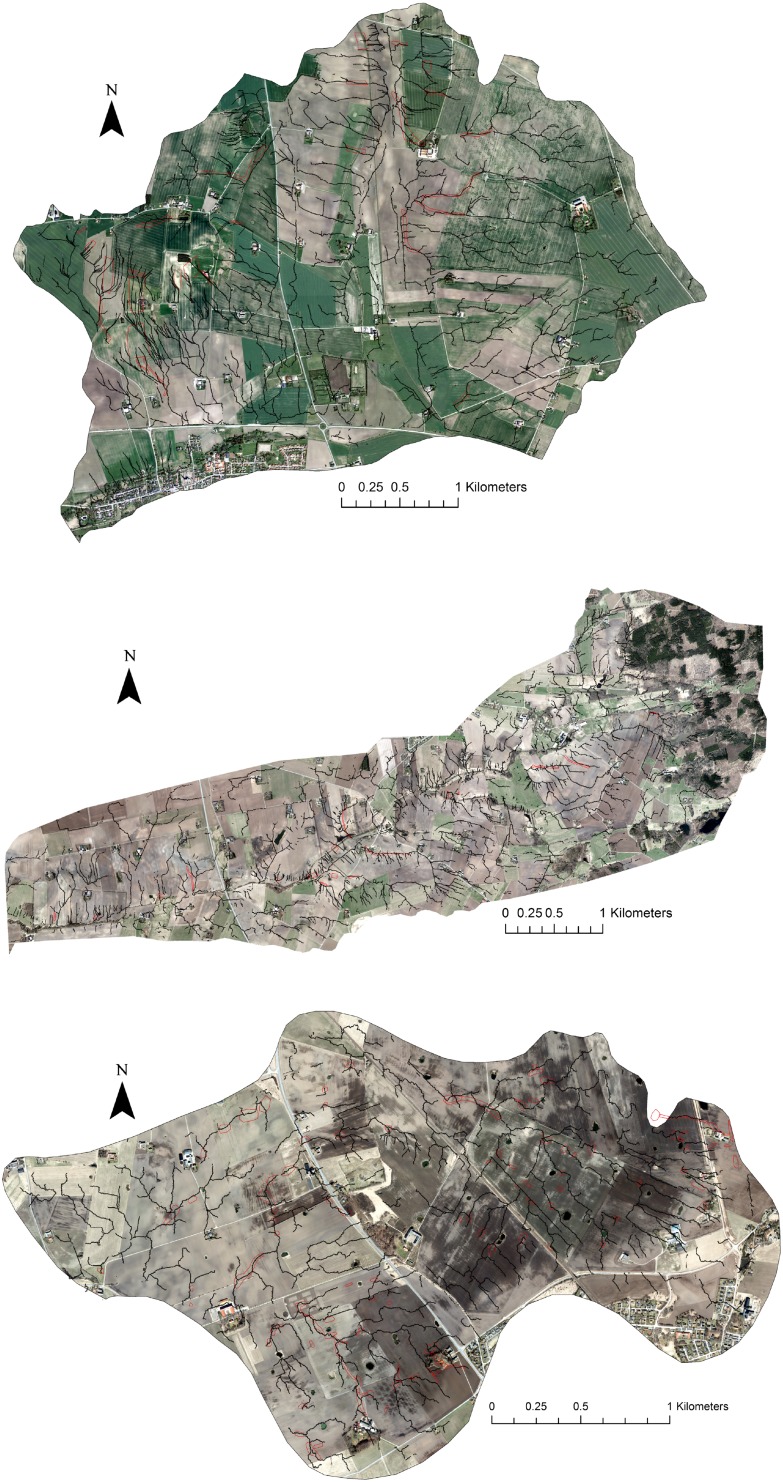

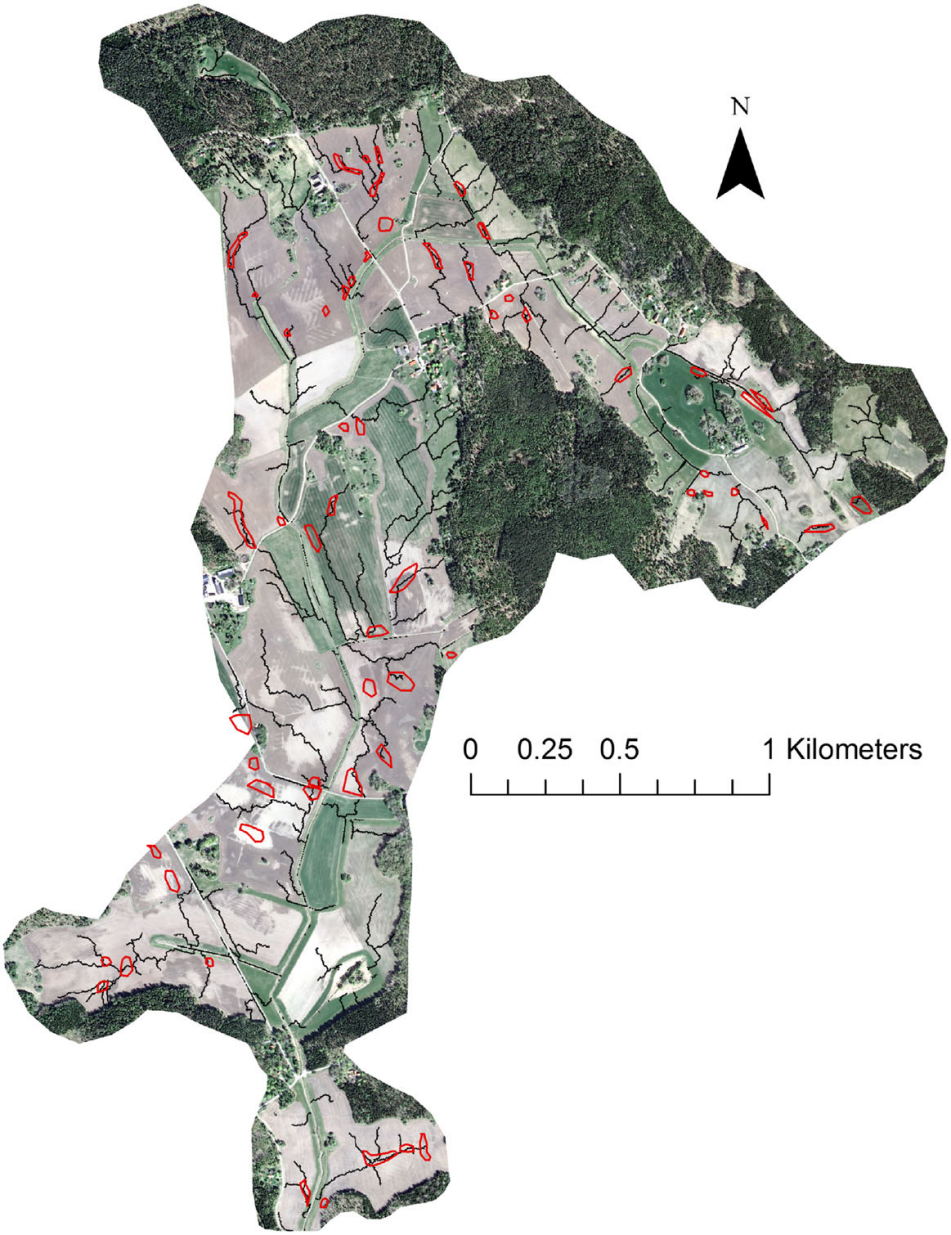
Observed (*red polygons*) and modelled (*black lines*) critical source areas of overland flow and erosion in four catchments in southern and central Sweden. Catchments order from *top* to *bottom*: Örstorpsbäcken, Långevadsbäcken, N33 and U8

The results of the field surveys, with identification and mapping of overland flow- and erosion-prone areas, are presented in Fig. 2 and Table 2. Overall, a low percentage (0.4–2.6 %) of the total area of arable land showed traces of overland flow and erosion. It should be noted that the observed traces of erosion and overland flow were event specific, meaning that the local conditions before and during the field surveys influenced the results. Permanent ley or other more protective crops prevent soil erosion, and traces of overland flow are difficult to discern. The observed CSAs were distributed across the study catchments (Fig. 2) and were usually small, with median sizes of 0.10, 0.13, 0.20 and 0.74 ha for catchment “N33”, “U8”, “Långevadsbäcken” and “Örstorpsbäcken”, respectively. The majority of the observed CSAs were long and narrow (Fig. 2), indicating that the probable cause of overland flow and erosion is more likely concentration of flow rather than precipitation intensity. Moreover, with few exceptions, the shape of the CSAs observed indicated that gully and rill erosion might be more frequent in the study catchments than was sheet erosion.

**Table 2:** Characteristics of observed critical source areas (CSAs) for overland flow and erosion

Catchment	CSAs (no.)	CSAs (ha)	Arable land (ha)	CSAs (% of arable land)	Median CSA area (ha)
Örstorpsbäcken	27	29.5	1402	2.1	0.74
Långevadsbäcken	17	4.3	1155	0.4	0.20
N33	101	14.7	569	2.6	0.10
U8	60	7.9	301	2.6	0.13

The spatial distribution of the modelled top 2 % of erosion-prone cells for each catchment is shown in Fig. 2, together with observed CSAs. The modelled erosion pathways intersected from 72 % (“U8”) to 96 % (“Örstorpsbäcken”) of the observed CSAs (Table 3). Field observations revealed that the model was unable to identify most of the CSAs where overland flow and erosion were caused by tramlines and compacted soil. Considering that the model is heavily reliant on topography as a first-order control on hydrology, the effects of soil compaction are not considered in the model, and therefore, failure to identify these CSAs is understandable. However, it is especially encouraging that the modelled flow pathways were in agreement with the observed long-narrow patterns of observed CSAs (Fig. 2). In addition, scattered CSAs observations were to a high degree connected by the modelled pathways, providing insights into the landscape connectivity.

**Table 3:** Comparison of critical source areas (CSAs) observed in field surveys and CSAs identified by modelling

Catchment	No. of observed CSAs	No. of observed CSAs intersected by modelled CSAs	Percentage of observed CSAs intersected by modelled CSAs
Örstorpsbäcken	27	26	96
Långevadsbäcken	17	14	82
N33	101	82	81
U8	60	43	72

Nevertheless, although only 2 % of top-ranked cells were highlighted, the model identified more erosion-prone areas than were observed in the field surveys. This was especially true for the two catchments (“Örstörpsbäcken” and “Långevadsbäcken”) where fewer observations of CSAs were made.

**Fig. 3:**
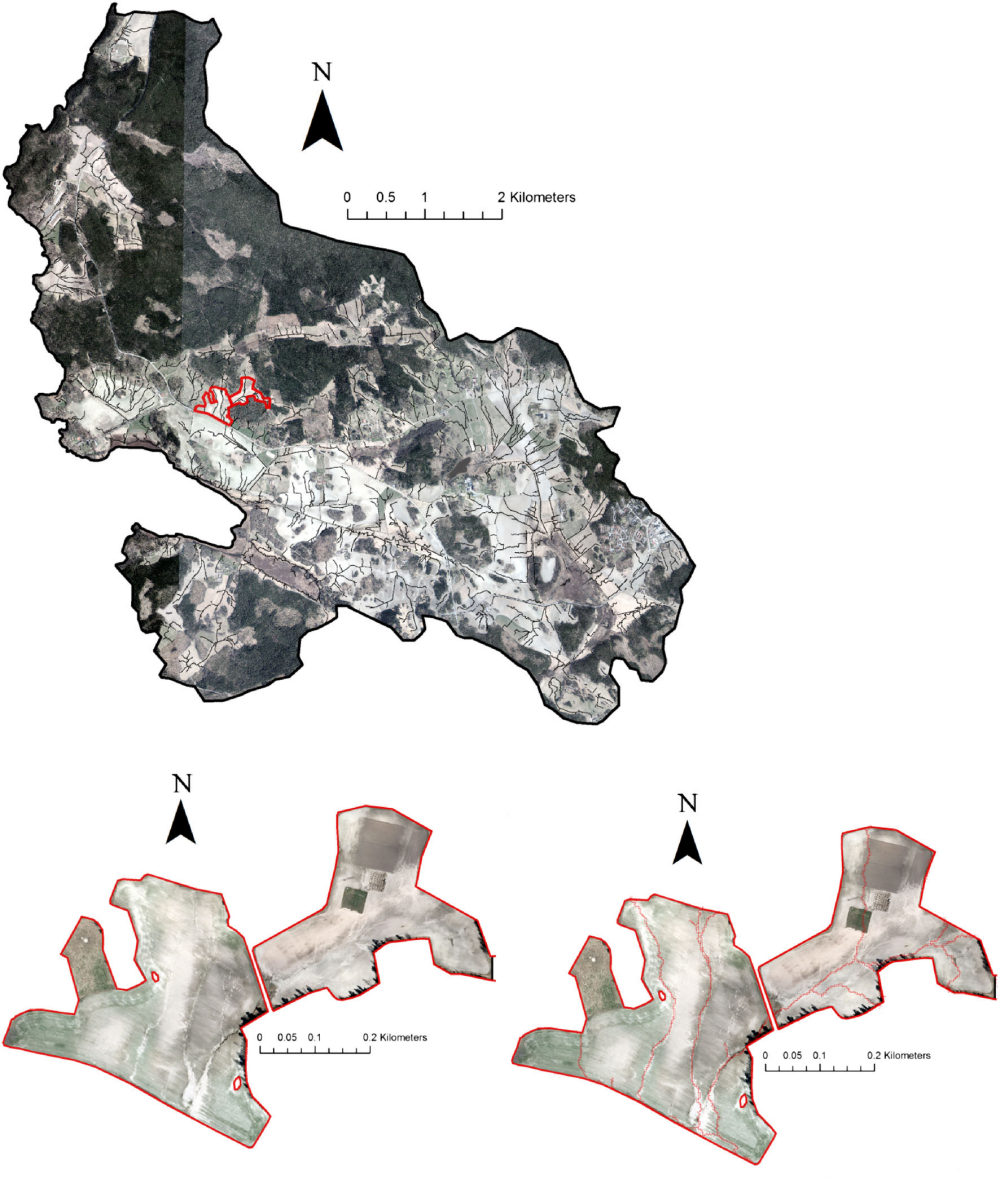
Modelled critical source areas (*black lines*) of overland flow and erosion in the “Krusenberg” catchment in central Sweden (*above*), and high-resolution aerial image showing erosion gullies (*lower left*) and modelled erosion pathways (*red lines*, *lower right*) in two vulnerable fields (*red polygons*)

**Fig. 4:**
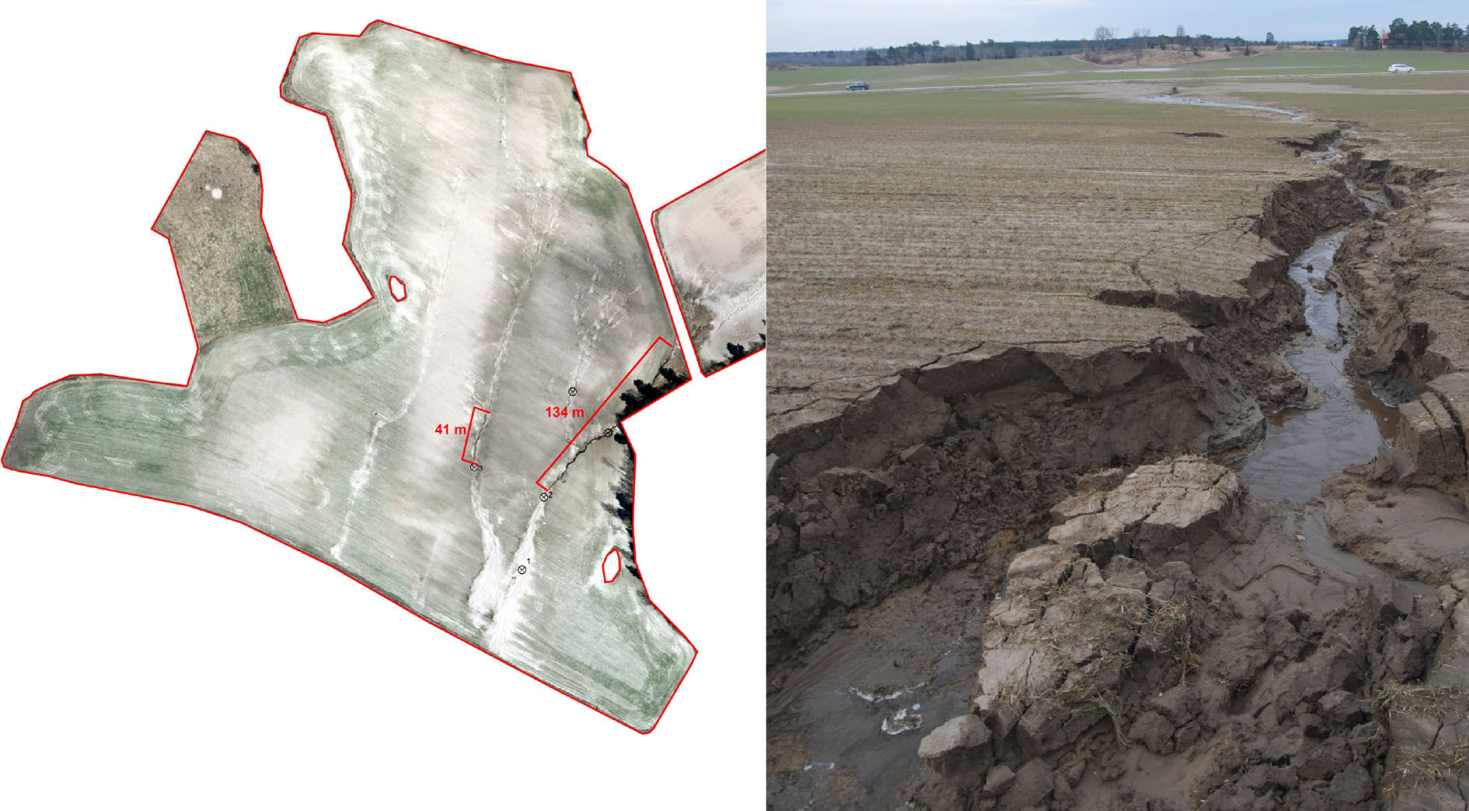
High-resolution aerial image showing visible erosion gullies in an erosion-susceptible field in the Krusenberg catchment and the positions of soil sampling points (*crossed circles*, *left*) and an image of a gully observed in April 2013 (*right*)

The modelling results in the “Krusenberg” catchment were used to identify fields at the highest risk, to which repeated field visits were made (Fig. 3). There were very few signs of overland flow and none of erosion during the first visit, in October 2012. However, frequent overland flow, ponding water and severe erosion were observed during the second visit, in April 2013. Severe gully erosion occurred on the study fields (Figs. 3, 4). Interestingly, although the “Krusenberg” catchment is dominated by clay soils, loamy sand was a dominant soil type on these particular fields.

The use of high-resolution data, primarily DEM, for modelling enabled very precise identification of the erosion pathways in the fields studied. Analysis of high-resolution aerial photographs confirmed that modelled erosion pathways and observed gullies showed a high degree of agreement (Fig. 4).

The total lengths of the two most severe gullies were 134 and 41 m. The width and the depth of these two gullies exceeded 1 m along their whole length. This means that at least 175 m^3^ of soil were mobilized and transferred during this single episode. The mobilized material was transported to the surface water inlet and then on to the downstream ditches.

The TP content in the topsoil samples from the same field varied from 483 to 543 mg P kg^−1^. The sample from the bottom of the gully showed a somewhat lower TP content (412 mg kg^−1^). The same applied for plant-available P (P-AL), with the concentrations in topsoil samples varying between 55 and 94 mg kg^−1^, whereas the P content was somewhat lower in the gully sample (49 mg kg^−1^), indicating moderate-to-high P content in the field. According to Swedish Board of Agriculture (2013), the optimal soil P content based on the P-AL method is between 40 and 80 mg kg^−1^. Using the typical soil density of soils from the same area and with similar texture, which is 1.4 g cm^−3^ (Wiklert et al. 1983), the estimated volume of mobilized soil (175 m^3^) and soil P content were used to estimate P mobilization. The average value (513 mg TP kg^−1^) for all samples was assumed to be representative for the field, and this resulted in a value of 121 kg TP mobilized from the whole field during this single episode. Dividing by the area of the field (12.3 ha), TP mobilization was almost 10 kg ha^−1^ during this single episode, without considering enrichment ratio.

Turbidity measured in water samples in the DESPRAL test varied between 134 and 243 nephelometric turbidity units, and the concentration of SS varied between 160 and 433 mg L^−1^. There was a strong relationship between turbidity and SS (*r* ^2^ = 0.81). Once again, the lowest values of both turbidity and SS were recorded for the sample from the bottom of the gully. The measured turbidity values were low compared to those reported in a previous study of five Swedish observation fields (range 781–2310 nephelometric turbidity units, Villa et al. 2014). However, the measured SS values were low to moderate in comparison to those in the fields studied by Villa et al. (2014), which ranged between 290 and 1381 mg L^−1^. Analyses of P constituents in water samples from the DESPRAL test showed that UP was the major P form (91–95 % of TP). The UP concentration varied between 0.24 and 0.71 mg L^−1^ and the DP concentration between 0.02 and 0.04 mg L^−1^. Interestingly, in spite of the lower mobilization of SS compared with other studies, the mobilized concentrations of UP and DP and the domination of UP were similar to those observed in previous studies (Withers et al. 2007; Villa et al. 2014).

## Discussion

In general, only a small percentage of the arable land studied here showed traces of overland flow and erosion, emphasizing the importance of accurate identification of CSAs as a precondition for appropriate placement and implementation of countermeasures aimed at reducing overland flow and erosion. The small size of the observed CSAs and their uneven distribution across the study catchments not only made this task difficult, but also indicated that generally applied countermeasures have little chance of achieving high cost effectiveness. For instance, grassed buffer strips along watercourses should probably be much wider at locations where overland flow and erosion do occur, but could also be narrower in the parts of the landscape where the risk of overland flow and erosion is low or non-existent.

The high spatial agreement of modelled erosion pathways and observed gullies confirms that modelling results can be used for high-resolution targeting of both monitoring activities and implementation of countermeasures. However, the episodic character of erosion processes requires not only the identification of spatial variations, but also targeted timing of monitoring efforts. In general, the periods of active overland flow and erosion are rather short under Swedish conditions, with duration varying from a few days to several weeks. However, as shown here, these episodes in relatively small parts of the fields can generate considerable loads to aquatic ecosystems. In the “Krusenberg” catchment, as much as 10 kg TP ha^−1^ were mobilized from a 12.3-ha field during one single episode. In fact, only small and identifiable parts of this field supplied the majority of the mobilized soil and associated P. Besides the direct losses from field to recipient waters, these episodes may also cause loading of the downstream ditch system where part of the mobilized material temporarily settles. This material (soil particles and P) can thereafter be re-mobilized from the ditch base during subsequent flow episodes that may in fact not cause direct losses from the fields, but are of sufficient magnitude to cause mobilization and transfer of previously settled sediment.

The DESPRAL tests indicated low to moderate soil dispersivity, as coarse-textured loamy sand soils are considered less erosive than medium- and fine-textured soils (Cerdan et al. 2010). Despite this, flow concentration in laterally concave parts of the field resulted in severe erosion, indicating that transport capacity, and not mobilization capacity, limited erosion processes. These results suggest that the control exerted by topography on hydrology and overland flow concentration might be a more important part of risk assessment than the inherent susceptibility of the soil to erosion. Consequently, under the conditions studied here, flow concentration caused severe rill and gully erosion on the fields dominated by coarse-textured soils. While the DESPRAL test has been reported to show good agreement in rainfall experiments, resulting in shallow flow in sheet runoff (Withers et al. 2007), it may not be representative of the more concentrated rill and gully erosion which generates coarse-textured sediment.

Despite using just the 2 % top-ranked cells, the model overpredicted the occurrence of CSAs as regards overland flow and erosion. One possible explanation is that all agricultural land was assigned same and rather high value of cover factor (*C*) representative for cereal crops. Such scenario may therefore be considered as a “worst-case” scenario suitable for general risk assessment. Giving consideration to the higher soil protection provided by, e.g. pastures and permanent grassland could improve this aspect of the modelling. However, the main aim with the risk modelling was to identify potential CSAs, which may or may not actually materialize during a given flow event or season. Thus, overprediction might even be desirable as a safety margin, although it is also important to stress that placement of countermeasures cannot rely solely on modelling results. Nevertheless, modelled risk maps are a rather effective tool in providing a comprehensive basis for discussions among farmers, researchers, advisors and authorities (Djodjic and Spännar 2012).

Paradoxically, the current system for environmental support within the rural development programme in Sweden may counteract the cost-effective placement of countermeasures. Environmental support is currently granted to farmers as compensation for loss of income and is not by any means related to the efficiency of the implemented countermeasure. This approach favours implementation of catchment-wide, generally implemented countermeasures, where site-specific topographical, hydrological and agronomic conditions are not considered. A relatively recently introduced countermeasure known as “adapted buffer strips” (Swedish Board of Agriculture 2014b) would probably be the most suitable BMP to address the patchy pattern of observed CSAs. However, the requirement that each adapted buffer strip should have an area of at least 0.25 ha is questionable, since many observed CSAs were smaller. This is especially true for the long-narrow CSAs with rill and gully erosion, where the protective effect could be achieved with smaller, well-placed strips. For instance, a 3-m-wide and 200-m-long adapted buffer strip (in total 0.06 ha) in the “Krusenberg” field with observed severe gully erosion would probably give a similar effect to much larger strips. Minimizing areas under BMP could also be crucial for farmers’ willingness to implement countermeasures, since they may be reluctant to give up productive land (Buckley et al. 2012). Furthermore, additional distinguishing between CSAs could be done based on their connectivity and proximity to surface water features. However, due attention here should be paid to man-made shortcuts such as surface water inlets, which are common in Sweden and serve as inlets for mobilized material to tile drainage systems.

An important benefit of the approach applied here is that the modelled results can be used to spatially connect observed scattered CSAs, which may help one understand the landscape processes and connectivity, and also better target problems that manifest downstream, but actually have their origins in upstream parts of the catchment. Although further development and refinement of the model is needed regarding quantification of erosion and P losses, the risk maps produced can be valuable for farmers, advisors and authorities. For farmers, modelled risk maps are often just well-needed confirmation of their own observations and experiences. For advisors and authorities, high-resolution risk maps offer an insight into local, site-specific conditions and a chance to systematize and concretize site-specific implementation of BMPs.

The approach applied here, with upslope contributing areas and calculations of flow accumulation paths, may also indicate parts of fields with higher soil moisture and even ponding water, which can trigger macropore preferential flow (Skaggs et al. 1994). However, the primary target is still the mitigation of lateral P losses via overland flow and erosion, and thus this approach will not be suitable in catchments where the main problem is vertical P leaching due to high degree of P saturation.

## Conclusions

There is a discrepancy between the spatially variable and episodic characters of P losses and current environment protection programmes designed and applied in rather a general way. Based on the results of this study, we can conclude

- Field surveys identified small parts of catchments (0.4–2.6 %) showing traces of overland flow and erosion.
- Reliable high-resolution identification of these CSAs is possible using distributed modelling based on high-resolution DEM in combination with soil and land use data.
- So far, targeting of CSAs is limited to fields with high soil P content. While this aspect is important from a sustainability point of view, it does not necessarily mean efficient reduction of P losses to aquatic environments.
- On the other hand, hydrologically active and connected areas may suffer from high P losses even if soil P content is low or moderate.
- Successful identification and risk mapping of these relatively small parts of the catchments provides an important discussion base for site-specific placement of countermeasures to reduce erosion and P losses whereby the most vulnerable parts of the agricultural landscape are targeted to increase cost effectiveness.

It is therefore important that legislation, environment protection programmes and agricultural subsidy initiatives recognize these new possibilities. One first step in that direction would be to change the approach of environmental protection programmes from compensation for loss of income to rewarding the cost effectiveness of implemented countermeasures.
